# The People’s Dental Journal

**Published:** 1863-01

**Authors:** 


### THE PEOPLE'S DENTAL JOURNAL,

*Edited and published by W. W. Allport, D. D. S, & S. T. Creighton, of Chicago. Quarterly. Price, fifty cents a year, in advance.*

This is a neat little journal, gotten up, as we understand it, for the purpose of giving instruction to the people in regard to the value and the care of their teeth. This a most laudable enterprise, one, the importance of which, we think, very few recognize, even among dentists; it is a subject upon which we have thought much, and spoken somewhat, and acted considerably for several years. It is one great instrumentality whereby the profession may be made to grow. Whether Dr. A. intends to confine the circulation of the People's Journal to his own patrons, or whether he proposes to furnish it to the profession, for distribution, to their patrons; we hope, the latter, by all means—it strikes us that an enterprise of this kind is a grand thing. Let the articles, for the most part, be brief, comprehensive, and to the point; let them be such, principally, as would suit the people of different localities.

We presume the Dr. would furnish them in quantities, to dentists, for distribution, for a trifle over the cost of publication.

We do not dictate, but only make these suggestions, hoping the Dr. may extend the enterprise. T.

